# Conjugation of microbial-derived gold nanoparticles to different types of nucleic acids: evaluation of transfection efficiency

**DOI:** 10.1038/s41598-023-41567-7

**Published:** 2023-09-06

**Authors:** P. Pourali, V. Dzmitruk, O. Benada, M. Svoboda, V. Benson

**Affiliations:** 1 Institute of Microbiology, Czech Academy of Sciences, Prague, Czech Republic; 2 Center of Molecular Structure, Institute of Biotechnology, Czech Academy of Sciences, Vesec, Czech Republic; 3 Institute of Analytical Chemistry, Czech Academy of Sciences, Brno, Czech Republic

**Keywords:** Microbiology, Nanoscience and technology

## Abstract

In this study, gold nanoparticles produced by eukaryotic cell waste (AuNP), were analyzed as a transfection tool. AuNP were produced by *Fusarium oxysporum* and analyzed by spectrophotometry, transmission electron microscopy (TEM), scanning electron microscopy (SEM), and energy dispersive X-ray spectroscopy (EDS). Fourier transform infrared spectroscopy (FTIR) and dynamic light scattering (DLS) were used before and after conjugation with different nucleic acid (NA) types. Graphite furnace atomic absorption spectroscopy (GF-AAS) was used to determine the AuNP concentration. Conjugation was detected by electrophoresis. Confocal microscopy and quantitative real-time PCR (qPCR) were used to assess transfection. TEM, SEM, and EDS showed 25 nm AuNP with round shape. The amount of AuNP was 3.75 ± 0.2 µg/µL and FTIR proved conjugation of all NA types to AuNP. All the samples had a negative charge of − 36 to − 46 mV. Confocal microscopy confirmed internalization of the ssRNA-AuNP into eukaryotic cells and qPCR confirmed release and activity of carried RNA.

## Introduction

The synthesis of nanoparticles that have potential application in the medical field is attracting attention. There are few types of nanoparticles used in this field because the nanoparticles used must exhibit minimal toxicity and high compatibility with the human body. Silver and gold nanoparticles (NPs) are among the most important nanoparticles in this field^1^. Silver nanoparticles (AgNPs) are generally used as antifungal and antibacterial agents, while gold NPs are mostly used as biosensors or as drug, ligand, or gene delivery agents. Gold NPs are also used for imaging and hyperthermia^2^.

There are various chemical and physical methods to produce these two types of nanoparticles (here referred as NB-AuNP). However, they have some disadvantages, such as the use of toxic chemicals or the generation of toxic byproducts during chemical production. The disadvantages of the physical method include time and high energy consumption, which usually results in polydisperse nanoparticles^3,4^. Therefore, a third approach was introduced, generally called biosynthesis or green or biological synthesis of nanoparticles. In this technique, different types of nanoparticles are produced by microorganisms through their enzymes or other reducing agents such as polysaccharides. The nanoparticles are produced inside or outside the cells (by components of cellular waste), which is called intracellular or extracellular method of NPs biosynthesis^5^.

The cell-derived synthesis is considered safe, and environmentally friendly. It can be easily scaled up and there are some studies on the production of various types of nanoparticles using different types of bacteria, fungi, algae as well as some plant strains^5^.

Gold NPs are among the best-known and most suitable gene delivery vehicles. In this technique, the surface functional groups of NB-AuNP are modified to adjust their ability to bind target molecules^6^. For example, NB-AuNP functionalized with cationic quaternary ammonium groups were able to bind to plasmid DNA^7,8^. In another study, NB-AuNP were functionalized with cetyltrimethylammonium bromide (CTAB)^9^ to achieve better electrostatic attraction (i.e. ionic bond formation) with siRNA. RNA can be conjugated to NB-AuNP via thiol or electrostatic interactions between the negative charge of RNA and the positive charge of the NPs. It has been shown that thiolated DNA and RNA can bind to NB-AuNP via thiol-gold interactions^10–13^. DNA with hexanethiol linkers could also bind to NB-AuNP^14^. siRNA-NB-AuNP were shown to be produced via a disulfide bond between NPs capped with PEG and N-succinimidyl 3-(2-pyridyldithio) propionate^15^.

Although the use of NB-AuNP as transporters for nucleic acids (NAs) has been widely explored^16–18^, to date there is no report demonstrating the ability of microbial-derived AuNP to enter cell cytoplasm and transfect different types of single- and double-stranded DNAs and RNAs. Therefore, the current study analyzed the capacity of these nanoparticles as a carrier for various NAs. Since one of the advantages of the microbial-derived nanoparticles is natural presence of reactive surface biomolecules, we attempted to achieve the conjugation of AuNP with different NAs with minimal manipulations and without the use of additional linkers or chemicals. In addition, the ability of such AuNP conjugates to transfect specialized eukaryotic cells was reviewed and discussed.

## Results

### Characterization the AuNP

#### Optical review

**Figure 1:**
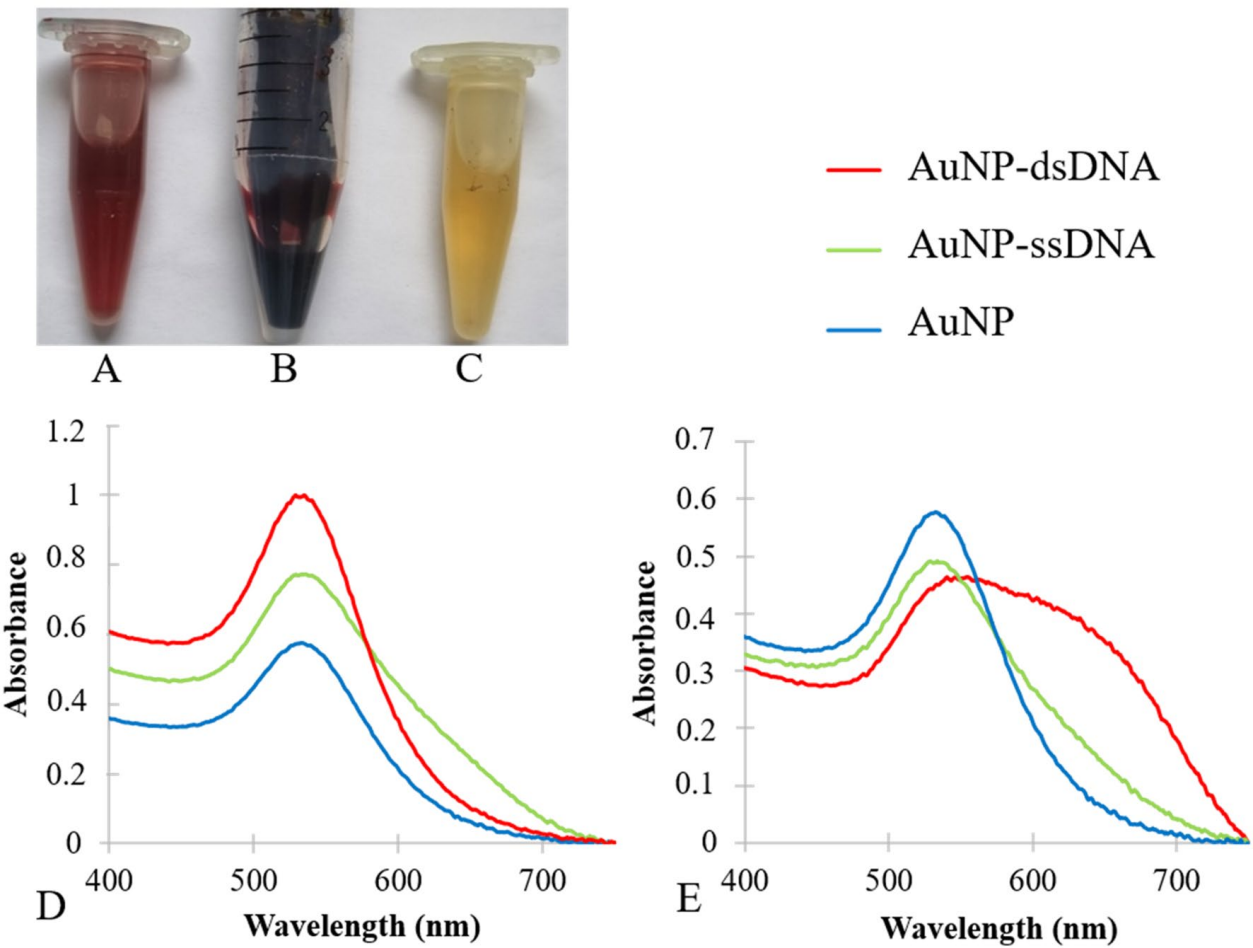
The produced AuNP, their spectrophotometry results before and after conjugation with NAs. (**A**) The color-changed supernatant after incubation with HAuCl_4_⋅3H_2_O (**B**), AuNP after washing, and (**C**) control supernatant. (**D**) AuNP-dsRNA and AuNP-ssRNA compared to AuNP. The maximum absorption peak of AuNP was 533 nm. (**E**) AuNP-dsDNA and AuNP-ssDNA compared to AuNP. The Supplementary Tables 1 and 2 show the absorbance values of each conjugate.

After 24 h of incubation with HAuCl_4_⋅3H_2_O, the color of the supernatant changed from yellow to crimson, in contrast to the stable yellow color in the untreated control sample. Figure 1 shows the changed color of the treated supernatant (Fig. 1A) in contrast to the control sample (Fig. 1C). Even after washing, the color of the sample containing AuNP did not change, indicating their stability in the aqueous environment (Fig. 1B). The darker color of the washed nanoparticles is due to the presence of the higher AuNP density.

#### Visible spectrophotometry for AuNP

The prepared AuNP had a maximum absorption peak at 533 nm. These nanoparticles were used for conjugation with dsDNA, ssDNA, dsRNA, and ssRNA molecules. The AuNP were diluted 1:8 to obtain the OD values below 1. Figure 1D shows the spectrum obtained.

#### Transmission electron microscopy (TEM)

**Figure 2:**
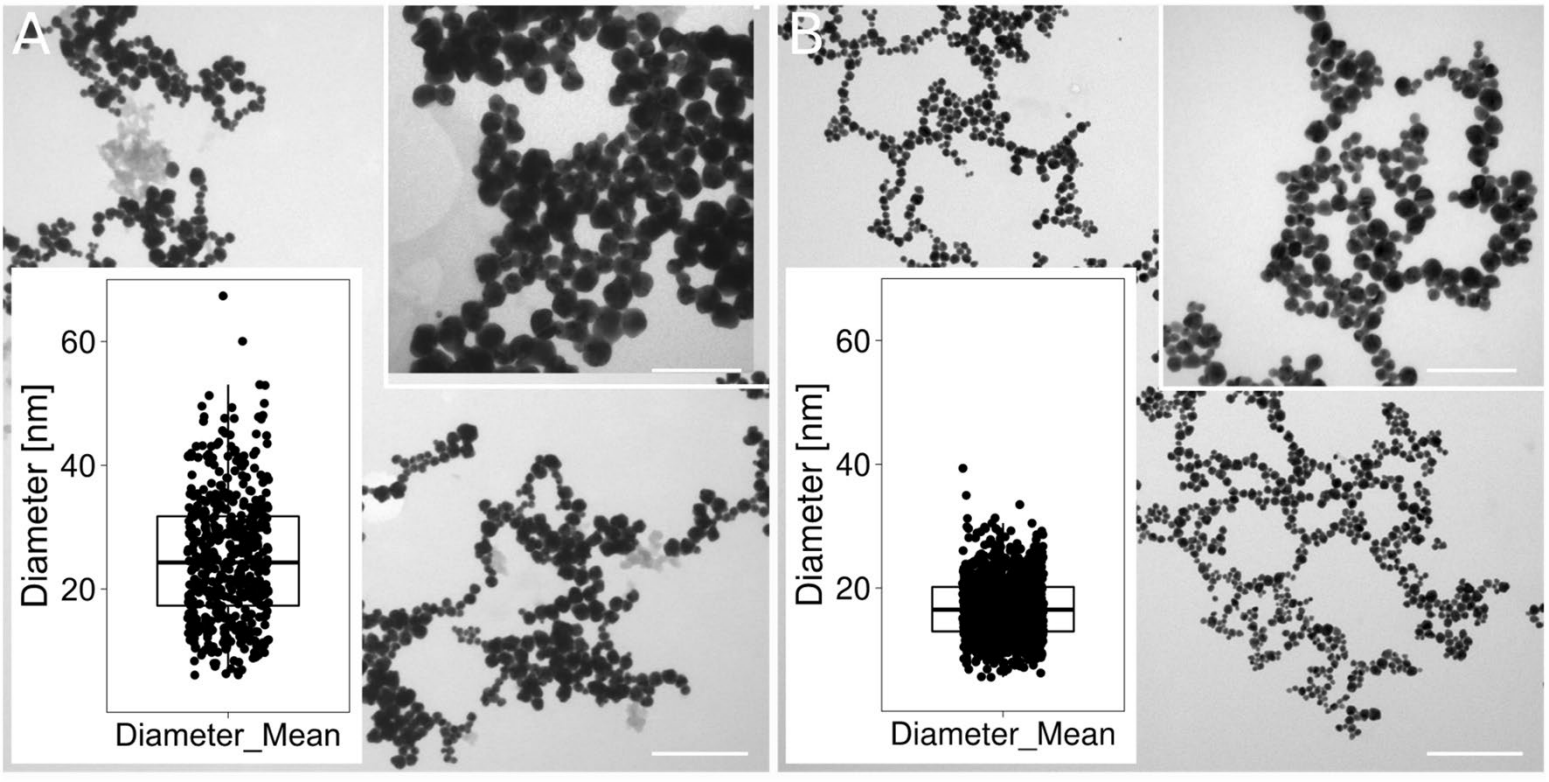
TEM images of AuNP with a round or polygonal shape and a size distribution of less than 40 nm. (**A**) and (**B**) show the same AuNP (before and after sonication) used for conjugation with different types of nucleic acids. Scale bars = 200 nm for the main images and 50 nm for the inserts. The inserts in the lower panels show the size distribution of the AuNP as a boxplot of the mean diameter of the particles shown in the corresponding panel.

The TEM images of the AuNP (Fig. 2) showed that the AuNP had round or hexagonal shapes and that there were distinct boundaries between the individual AuNP, which was due to the presence of capping agents that prevented the aggregation of the AuNP^19^. The average size of the AuNP was 25 nm.

#### Scanning electron microscopy (SEM) and energy dispersive X-ray spectroscopy (EDS)

**Figure 3:**
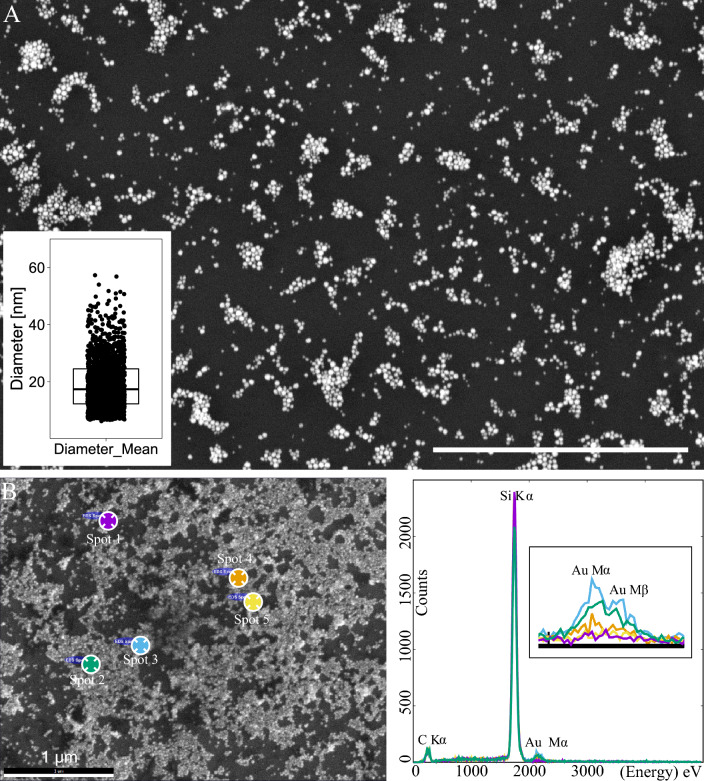
SEM and EDS analysis of AuNP deposited on glow discharge activated silicon wafers and coated with 10 nm carbon. (**A**) SEM Analysis of AuNP used for conjugation with different types of NAs; a CBS detector at 3 kV. The insert shows the size distribution of AuNP as a boxplot of the mean diameter of the particles. (**B**) The EDS analysis of the same sample at 10 kV; an ETD detector image with the marked positions of the spectrum acquisition on the left and the corresponding spectra on the right. The insert in the spectra part of B shows the magnified region of Au Mα and Mβ lines with a noticeable difference of the spectra from the background (spots 1 and 5) and the AuNP (spots 2, 3, and 4). Scale bars = 1 µm.

Figure 3 shows the results from SEM and EDS of microbial-derived AuNP.

#### Zetasizer analysis before conjugation with NAs

The measurements were performed at least three times and Table 1 shows the results of the particle size and zeta potential analyses. The AuNP had acceptable size and zeta potential (Table 1).

**Table 1:** Hydrodynamic diameter and zeta potential.

Analysis type	Repeats	AuNP	AuNP-ssRNA	AuNP-ssDNA	AuNP-dsRNA	AuNP-dsDNA
Diameter (nm)	I	38.0	28.0	38.0	49.0	33.0
	II	38.0	38.0	38.0	33.0	33.0
	III	32.0	32.0	32.0	33.0	33.0
	IV	32.0	32.0	32.0	41.0	33.0
	V	38.0	32.0	38.0	33.0	33.0
	Mean	39.2	32.4	35.6	37.8	33.0
	SD	4.5	3.2	2.9	6.4	0.0
Zeta-potential (mV)	I	− 38.2	− 38.1	− 39.3	− 43.5	− 41.1
	II	− 34.4	− 42.7	− 39.2	− 48.1	− 40.0
	III	− 36.8	− 37.2	− 38.8	− 46.7	− 41.9
	Mean	− 36.8	− 39.3	− 39.1	− 46.1	− 41.0
	SD	1.5	2.4	0.2	1.9	0.8

AuNP were used for conjugation with different types of NAs.

#### Graphite furnace atomic absorption spectroscopy (GF-AAS)

Considering the determined Au concentration in different diluted samples and the respective dilution factors, the AuNP sample which was used for conjugation study contained 3.75 ± 0.2 µg/µL of AuNP.

### Evidence of the conjugation of AuNP to NAs

#### Visible spectrophotometry for AuNP-NAs

All single- and double-stranded RNA and DNA molecules were stable, and only minor differences were observed between their maximum absorption peaks. Figure 1 shows the differences in the spectra. For the RNA conjugates (Fig. 1E), the maximum absorption peak of the AuNP was at 533 nm, whereas for the AuNP-dsRNA, two peaks occurred at 529 and 535 nm, and for the AuNP-ssRNA, three points occurred at 533, 535, and 536 nm. For DNA conjugates (Fig. 1F), the maximum absorption peak for AuNP was at 533 nm, while for AuNP-dsDNA it was 555 nm and for AuNP-ssDNA two points were at 529 and 534 nm. To facilitate comparison, the absorbance values of each spectrum are listed in the Supplementary Tables 1 and 2. Due to the differences in the nucleotide sequences and fragment lengths of dsDNA, ssDNA, dsRNA, and ssRNA, the maximum absorbance peaks shifted to higher or lower wavelengths after conjugation of the different NAs with AuNP. A completely different spectral curve was observed in the AuNP-dsDNA sample. That sample possessed the longest maximum absorption wavelength (555 nm), and as can be seen in Fig. 1F, this conjugation resulted in changes in the spectrum, indicating that the AuNP were likely unstable after conjugation with dsDNA.

#### Electrophoretic mobility of conjugates

**Figure 4:**
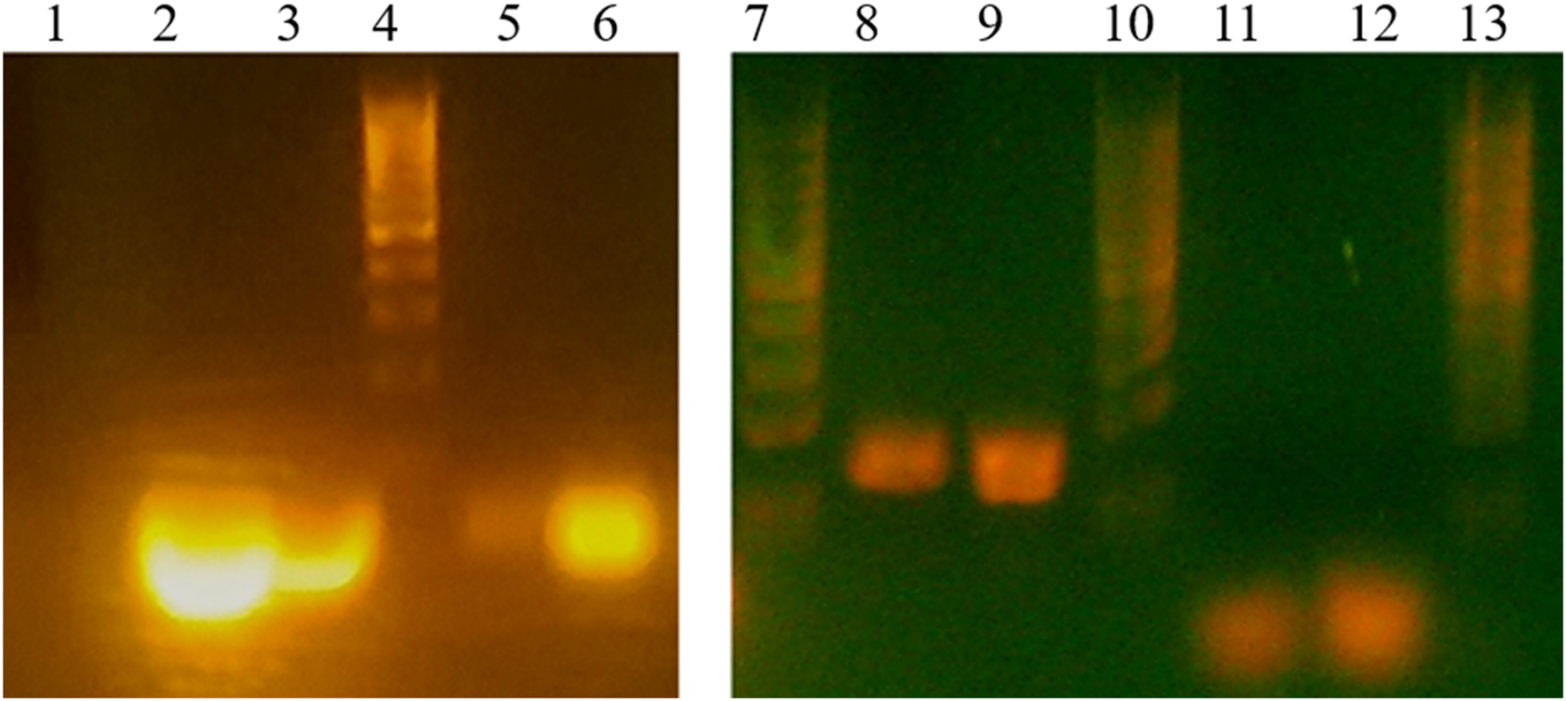
Electrophoretic mobility. Lanes 4, 7, 10 and 13 correspond to the MassRuler low range DNA ladder. Lane 1 is the control AuNP, lane 2 control dsRNA, lane 6 AuNP-dsRNA, lane 3 control ssRNA, lane 5 AuNP-ssRNA. Lane 9 control dsDNA, lane 8 AuNP-dsDNA, lane 11 control ssDNA, and lane 12 is AuNP-ssDNA.

Agarose gel electrophoresis was performed for different AuNP-ssRNA, AuNP-dsRNA, AuNP-ssDNA, and AuNP-dsDNA conjugates at different concentrations. Migration delay of conjugates compared to controls was observed for all samples, which was evidence of successful conjugation. The final concentration of 10 µmol NA gave the best result for all samples and that concentration was used in the experiments if not stated otherwise. Figure 4 shows a representative electrophoresis of the conjugates. The data demonstrate that both double-stranded and single-stranded DNA and RNA are directly linked to AuNP.

#### FTIR

**Figure 5:**
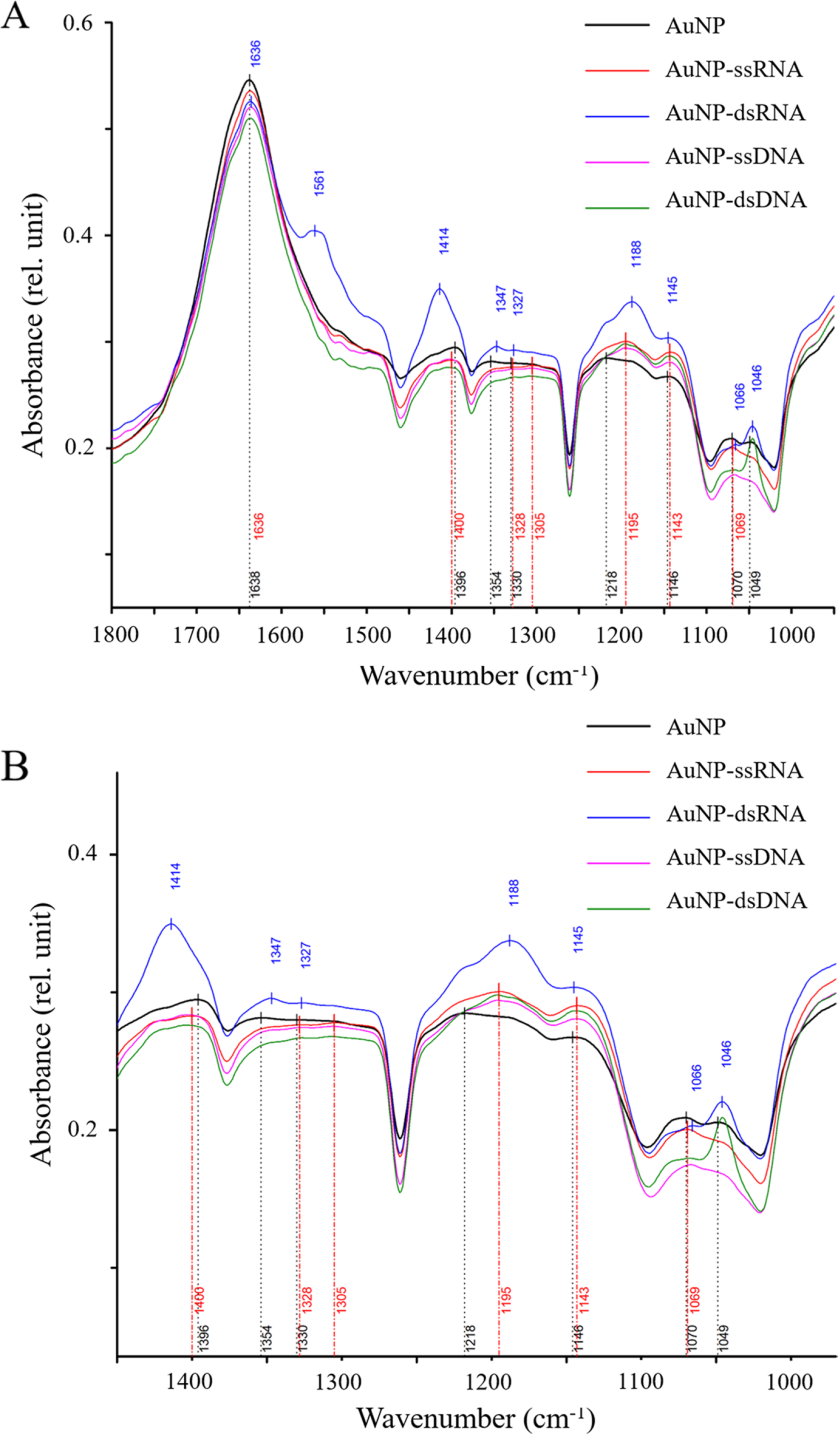
IR Spectra of all AuNP conjugates compared to AuNP as control. (**A**) The spectra are in the range 900–4000 cm^−1^. (**B**) Detailed spectra from the range of 900–1800 cm^−1^.

The IR spectra of all AuNP-NAs and AuNP as control have a wide and very broad peak centered at 3300–3330 cm^−1^, which essentially corresponds to the O–H, N–H, and C–H stretching of AuNP and may include the O–H stretching signal of the solvent water (Fig. 5A). There are also 3 peaks that appear as the signal of C-H alkane stretching bands in all samples at 2943, 2884, and 2821 cm^−1^ (Fig. 5A). The amide I peak (C=O stretching) is at 1638 cm^−1^ for AuNP, while it is shifted to 1636 cm^−1^ for AuNP with NA conjugates, indicating increased mass of the compound. For better understanding, Fig. 5B shows the spectra obtained from 900 to 1800 cm^−1^. As it can be seen in Fig. 5, the characteristic part of the IR spectrum for the NA is between 900 and 1300 cm^−1^ (Fig. 5B). Since the AuNP naturally possess different biomolecules on their surface, we can detect background peaks in the control AuNP sample.

For AuNP-dsRNA, additional peaks were observed at 1561 and 1414 cm^−1^. Peak at 1561 cm^−1^ can be identified as an amide II band associated with N–H bending and C–N stretching, 1414 cm^−1^ might correspond to additional C–H stretching (Fig. 5B). Identification of the main characteristic peaks of AuNP loaded with NAs compared to AuNP reveals 2 common peaks at 1145 and 1195 cm^−1^. The symmetric stretching band of phosphates is ∼1090 cm^−1^ for all NA forms [Contribution of RiboNA (RNA) to the FTIR Spectrum of Eukaryotic Cells], which could confirm the presence of NAs in the studied AuNP. The most prominent peak for double-stranded NAs coupled to AuNP is at 1046 cm^−1^, which could correspond to the C–O strength of ribose and deoxyribose (Fig. 5B).

#### Zetasizer analysis of AuNP and their conjugates with NAs

The measurements were performed at least three times and Table 1 shows hydrodynamic diameter (size) and zeta potential of the AuNP before and after conjugation with various single- and double-stranded RNA and DNA molecules.

The results from Table 1 show that the average size of AuNP decreased after conjugation with NAs in all samples and the zeta potential became more negative in all samples due to the negative charge of NAs. The highly negative values of the zeta potential suggest great stability of the conjugates.

### In vitro cellular assays

#### Analysis of AuNP sterility

The optical assessment of the sample sterility showed that the AuNP were sterile and there were no visible bacterial or fungal colonies on the surface of the agar plates after the incubation period (data not shown).

#### MTT assay of toxicity

Before analyzing the cellular uptake of the AuNP-NAs, the toxicity of the AuNP in the NIH/3T3 and 4T1 cell lines as normal and cancerous cells was checked using the MTT assay. Based on the results from GF-AAS, the maximum amount of AuNP equaled to 187.5 µg and the minimum amount of AuNP equaled to 0.183 µg. The results showed no toxic effect for either cell line when compared to the control wells (cells with no exposure to AuNP).

#### Internalization of conjugates into cells

**Figure 6:**
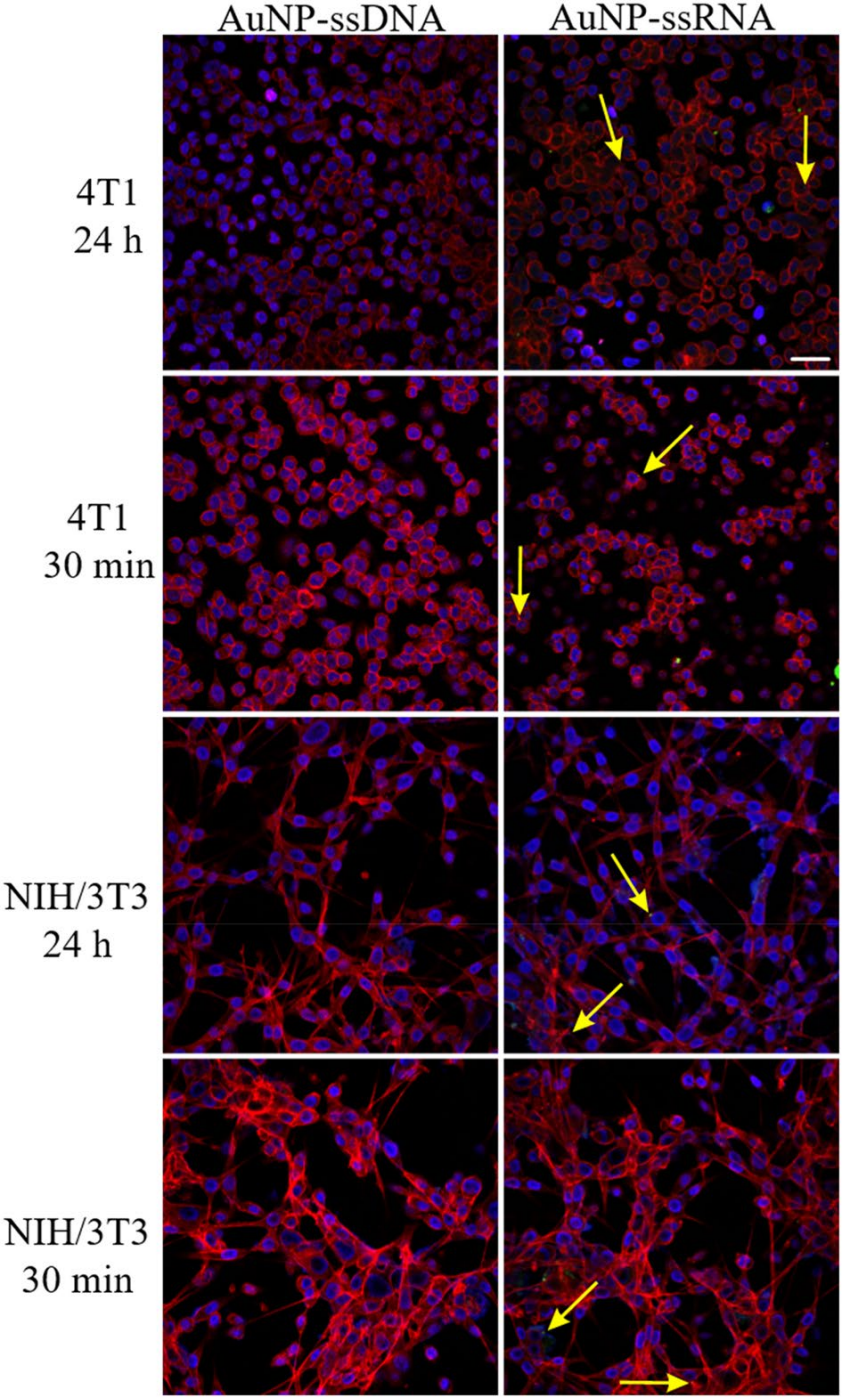
Internalization of AuNP-ssDNA- and AuNP-ssRNA- by 4T1 and NIH/3T3 cells. The cells were incubated with conjugates for 30 min or 24 h. Yellow arrows point to the internalized complexes. Cell nuclei are in blue, F-actin filaments are in red, and AuNP-carried NAs are in green. Scale bar = 20 µm.

We present internalization of two of the four NAs in two different (non-phagocytosing cells). Both, NIH/3T3 and 4T1 cells were incubated with the AuNP-Alexa Fluor 488-ssRNA or AuNP-Alexa Fluor 488-ssDNA conjugates for 30 min or 24 h. After incubation, the cells were analyzed under confocal microscopy to evaluate the cellular uptake of the conjugates. Figure 6 show representative images for the different conjugates and cell lines.

Confocal microscopy showed that only the AuNP loaded with ssRNA could enter the 4T1 cells. The conjugates were internalized very rapidly (within 30 min-incubation) and they were detectable in the cells after 24 h. The same phenomenon was observed in the NIH/3T3 cell line, but there was less overall positive signal than in 4T1. This could be due to the higher uptake activity of the cancer cells (4T1) in contrast to the immortalized fibroblasts (NIH/3T3).

#### Release of ssRNA cargo from AuNP detected by qPCR

**Figure 7:**
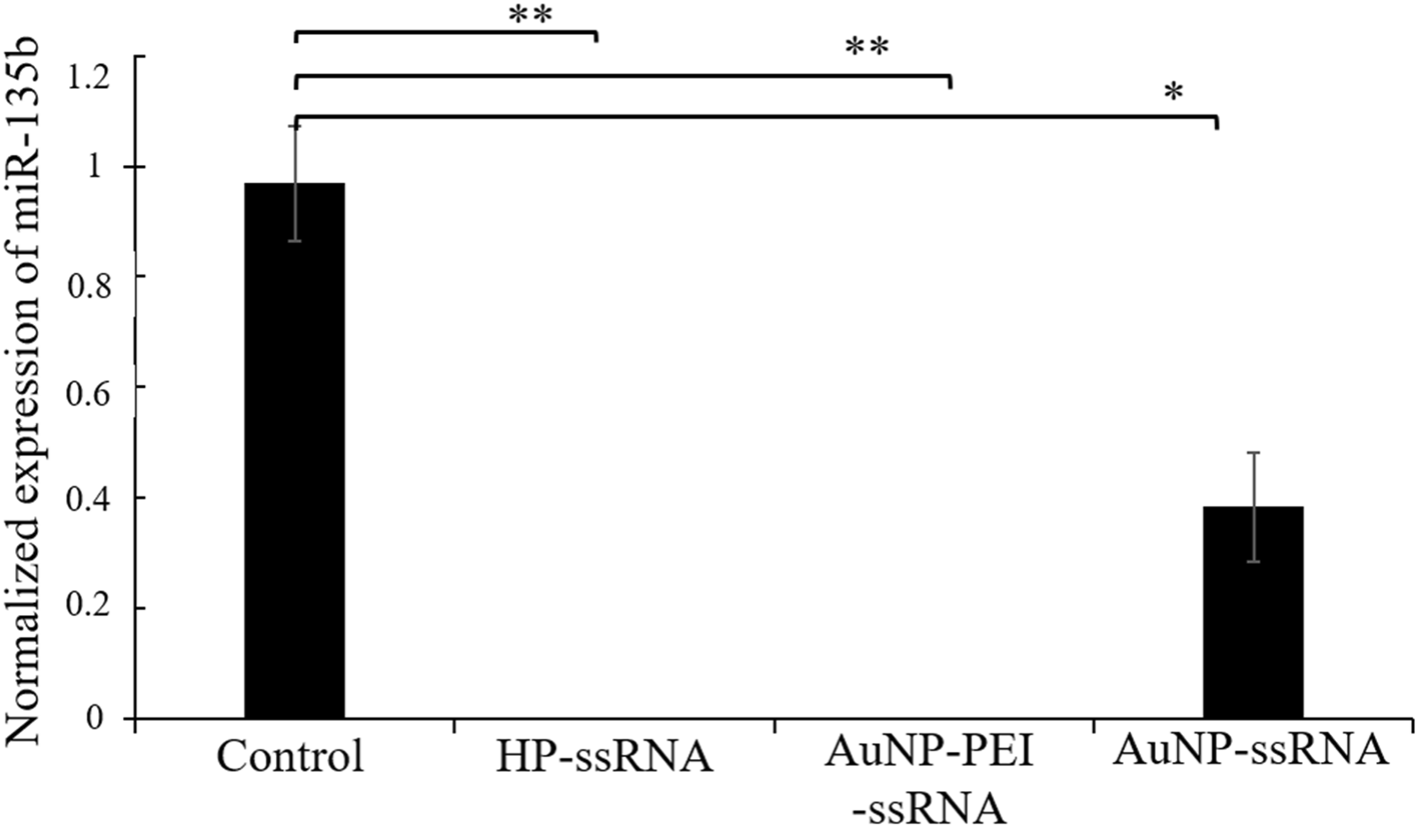
qPCR-based detection of miR-135b level in 4T1 cells incubated with different conjugates. Control cells incubated only with PBS, cells incubated with HP-ssRNA, AuNP-PEI-ssRNA, and AuNP-ssRNA, respectively. Values of p ≤ 0.05 (*) and p ≤ 0.01 (**) were considered statistically significant between groups.

Since the microscopy showed that ssRNA (inhibitory RNA targeting miR-135b; antimiR-135b) was successfully transfected into the 4T1 cells, we performed qPCR analysis to evaluate its functionality to reduce the miR-135b level in cell cytoplasm. Figure 7 shows the normalized levels of miR-135b in different samples. We used two positive controls of transfection, HP-ssRNA and AuNP-PEI-ssRNA that both possess high capacity in RNA transfection^20^ and, as expected, they exhibited high rate of antimiR-135b transfection proved by complete elimination of miR-135b (p-value < 0.01 in cells incubated with HP-ssRNA and AuNP-PEI-ssRNA). A significant decrease (p-value < 0.05) in miR-135b expression was detected in 4T1 cells incubated with antimiR-135b linked to AuNP too. The relative levels of reference control miR-16 reflecting the amount of microRNA fraction in each sample are shown in Supplementary Fig. 2. Mir-16 was used for miR-135b level normalization.

## Discussion

Successful transfection of functional nucleic acids strongly relies on chosen delivery vehicle^21^. There are different delivery systems such as cationic phospholipids (e.g., liposomes), cationic polymers (e.g., dendrimers, and polyethylenimine), various peptides, and viral vectors (e.g., herpesviruses, adenoviruses, retroviruses). Each of them has its own advantages and disadvantages^22^. The NA vector must have some characteristics, such as low toxicity, ability to bind and protect the introduced NA, disrupt the endosomal membrane, and deliver the NA to the cytoplasm and/or nucleus. If the vehicle can also target a specific cell type, it is superior.

AuNP are considered as NA delivery agents. However, the ability of microbial-derived AuNP to bind to various NAs, but also the ability to transfect the cells and deliver the specific NA molecule into the cytoplasm is still completely unknown. The main advantage of the microbial-derived AuNP, besides their low toxicity and biocompatibility, is that they can bind directly to the NA molecules, eliminating the additional steps to change the surface charge like in the NB-AuNP.

In this study, the AuNP were successfully prepared using *F. oxysporum* and analyzed by various methods. Although the TEM results showed that the average size of the AuNP was 25 nm, the zetasizer/DLS showed 39 nm particles. According to Maguire et al., the average NPs sizes measured by TEM differ from those obtained by zetasizer/DLS because DLS measures hydrodynamic diameter and TEM measures diameter of nanoparticle core^23^. We experienced that in our previous research^24^ as well as the current study.

The used method for conjugation of the AuNP with different types of NAs was simple adsorbtion overnight at 4 °C (cold temperature was used to decrease the chance of NA degradation by nucleases). Although both of the AuNP and NAs had negative charge, the conjugation occurred. This was due to the capping agents on the surface of AuNP, which can bind with the different reactive groups of DNA and RNA. Previously, some studies showed that the capping agents consist of microbial proteins secreted into extracellular ambiance^25^. These molecules coat the surfaces of the AuNP during their formation and prevent them from aggregating^26^. The capping agents are also responsible for the overall negative surface charge of the AuNP used in this study. Theoretically, conjugation of the AuNP with the NAs, which have the same negative charge, is impossible due to the repulsion between the phosphate groups of the NAs and the negative surface charge of the capping agents. However, there is a report showing that ssRNA can bind to the negatively charged NB-AuNP, because the polar ssRNA can fold under these conditions and bind to the negatively charged nanoparticles through its amine groups. On this basis, there are reports using NB-AuNP as an ultrasensitive biosensor to detect specific ssRNA or ssDNA molecules^27,28^.

It has been shown that ssDNA can attach to the negatively charged NB-AuNP, further enhancing the repulsion between the NB-AuNP and preventing aggregation^29^. In contrast, dsDNA could not act in a similar manner due to the repulsion between the DNA phosphate scaffold and the surfaces of the NB-AuNP^29,30^. According to the spectrophotometry results of each conjugate in the current study, the NB-AuNP were likely unstable after conjugation with dsDNA which might be due to the structure of the dsDNA.

Similar study with ssRNA (HCV RNA detection in sera) was performed by Shawky et al.^28^. To our knowledge, there are other ssRNA molecules in the serum that could attach to the surface of AuNP and stabilize them, and the application of AuNP as an accurate biosensor requires further research. In the current study, we demonstrated that both single-stranded and double-stranded RNA and DNA molecules could attach to the surface of AuNP. Probably, all the four types of NAs could attach as polar molecules to the oppositely charged polar side chains of the polypeptides or amino acids serving as AuNP capping agents.

FTIR was performed to detect the formation of AuNP-NAs. From the FTIR results, it is obvious that both types of NAs were conjugated to the AuNP without additional linkers. There are studies on the use of NB-AuNP as carriers for different types of NAs. Covalent binding of NAs to such NB-AuNP and transfer of antimiR and small interfering RNAs (siRNAs) have already been studied^31–33^. There, thiol-modified RNAs were used to attach to the NB-AuNP. Non-covalent binding of NAs (DNA or RNA) to the positively charged NB-AuNP has also been investigated^7,17^. For example, polyethylenimine (PEI) coat changes the surface charge of the NB-AuNP to obtain positively charged particles^18^. Another option is the use of amino acids to tailor the functionality of the NB-AuNP. It has been shown that the NB-AuNP functionalized with cationic amino acids can bind to dsDNA^34^. Although the AuNP possess a net negative charge, due to their capping agents, which are mostly polypeptides or amino acids^35^. Thus, that AuNP could possibly attach the negatively charged NAs through the cationic amino acids within AuNP capping agent. In this study, both types of ssRNA and dsRNA as well as both types of ssDNA and dsDNA were directly adsorbed onto the AuNP.

We have already shown that the AuNP are able to conjugate directly with various drugs^36–38^ as well as enzymes^39^ due to their specific surface capping agents^24^, which is the most important advantage of the microbial-derived AuNP in comparison to the NB-AuNP. In this study, we showed that conjugation of AuNP with various NAs is possible with minimal manipulations and without the use of additional linkers or chemicals.

After conjugation of AuNP with different types of NAs and their characterization, the conjugates were tested in vitro. The first step was to evaluate the toxicity of each of the conjugates in cell culture. Although we have shown in our previous work that there were some dose-dependent toxic effects of the AuNP^5,40^, we did not find any toxic effects of the AuNP on different cell lines in this study (the results are represented in Supplementary Fig. 1 and Supplementary Table 3 for NIH/3T3 and 4T1 cell lines). This could be due to lower concentrations of nanoparticles or the use of three washing processes that removed residual impurities from the AuNP. The impurities in our previous studies could lead to toxicity in cell culture. The shape, size, and surface charge of the nanoparticles may also differ^41^. The type of microorganism used to produce nanoparticles affects the size and shape, which may affect their toxicity^26^. Thus, the results of this study suggest that the AuNP are biocompatible after three washes and can be used directly in in vitro assays.

NAs transfection to specialized nonphagocytic cells requires their transfer across the plasma membrane^42^ and data of two of the NA samples (i.e., ssNAs) are shown because, according to the spectrophotometry results, the ssDNA and ssRNA conjugates exhibited good stability and their peaks were closest to the control AuNP. Regarding the AuNP-ssDNA, although AuNP bound to both ssDNA and dsDNA, there were no AuNP-ssDNA complexes in either cell line, suggesting that these complexes had a very low transfection rate in contrast to AuNP-ssRNA. Hence, the differences in the nature of the ssRNA in contrast to ssDNA due to the presence of uracil instead of thymine^43^ as well as DNA folding might affect their cellular penetration when loaded onto AuNP. We analyzed AuNP-dsRNA and AuNP-dsDNA conjugates too (data not shown) but the conjugates exhibited minimal cell internalization. Therefore, the AuNP-ssRNA was the most promising conjugate and we focused on it within the following experiments.

In the last step, qPCR proved that inhibitory ssRNA (antimiR-135b) was successfully transfected into the 4T1 cells and released into cell cytoplasm to reduce its target miR-135b level. This means that the AuNP attached the NA molecule, transfected the cell culture, and the transfected NA retained its activity to regulate gene expression after internalization. In the future, we plan to conjugate the AuNP with a cancer cell specific structure (such as transferrin, folate, or antibody) to provide vector for targeted intracellular delivery.

## Conclusions

So far, from the group of biological vectors for the transport of NAs, the viral vectors are the most popular but they possess disadvantages, such as the induction of inflammation, immunogenicity, and potential carcinogenicity^44–46^. The microbial-derived AuNP represent an alternative to the viral vectors. They are less toxic and non-immunogenic, but further studies regarding their efficacy and safety are needed. This is the first report showing the ability of AuNP directly attach NAs and penetrate the cytoplasmic membrane. The confocal microscopy showed that the ssRNA-loaded AuNP entered the cancerous cells as well as immortalized fibroblasts without detectable damage to the cell membrane proved by the MTT assay. In addition, it was obvious that the ssRNA-loaded AuNP were able to penetrate the cells more effectively, unlike the AuNP-ssDNA, which might be due to their structural differences. The qPCR results confirmed that the biological AuNP not only attached to the ssRNA (antimiR-135b) and transfected it to the cells, but also the antimiR-135b escaped to the cell cytoplasm, bound to and knocked down the level of target miRNA. Further analysis is required to understand the mechanism of cell penetration (i.e., endocytosis, direct penetration, etc.) as well as endosomal escape and cargo release from the AuNP, which we also plan to perform in future.

## Methods

### AuNP production

*Fusarium oxysporum* was used for the extracellular production of AuNP. The fungus was acquired from the Culture Collection of Fungi of the Charles University (CCF 3732, Czech Republic). After cultivation in Sabouraud Dextrose Broth (SDB, Sigma Aldrich, Prague, Czech Republic) at 30 °C and 150 rpm for 4 days, the supernatant of the culture was obtained by centrifugation at 8000 rcf for 10 min. 100 µL of 1 molar solution of HAuCl_4_⋅3H_2_O (Sigma Aldrich, Prague, Czech Republic) in ddH_2_O was added to 100 mL of the supernatant and its pH was adjusted to 7. The dispersion was incubated at 37 °C under shaking conditions (200 rpm) for 24 h. The final concentration of HAuCl_4_⋅3H_2_O was 1 mmol and a negative control flask contained sterile SDB with the same concentration of gold ions was incubated with the others^36^.

### Washing AuNP

If the AuNP were produced after the incubation period, the color of the supernatant would change, unlike the control. Since the flask contained AuNP as well as other contaminants of the culture medium such as Au ions and other substances secreted by *F. oxysporum*, the mixture was washed with RNase-free ddH_2_O (pH 7.4, Qiagen, Hilden, Germany) and centrifuged at 22,000 rcf for 30 min three times. The pellet was dissolved in 1000 µL RNase free ddH_2_O and used for further analysis^5^.

### Characterization of the AuNP

#### Optical review

The first sign of AuNP production due to their localized surface plasmon resonance (LSPR) is the color change of the reaction mixture from yellow to different color ranging from red, pink, or green to blue based on the shape and size of formed AuNP^38^.

#### Visible spectrophotometry for AuNP

Due to LSPR, the prepared AuNP have a maximum absorption peak between 500 and 550 nm. Therefore, the optical density (OD) of the produced AuNP was measured using Nanodrop spectrophotometer (Thermo Fisher Scientific, Massachusetts, USA). The wavelengths used were between 400 and 750 nm and ddH_2_O was used as a blank. In the measurement, AuNP were diluted with ddH_2_O to obtain OD below 1^38^.

#### Transmission electron microscopy (TEM)

Philips CM100 TEM (Philips EO, Eindhoven, The Netherlands) equipped with a Veleta slow-scan CCD camera (EMSIS, Muenster, Germany) was used to analyze AuNP sample. For this purpose, 5 µL of the AuNP sample was applied onto the Formvar/carbon-coated 300 Mesh copper grid activated by glow discharge (1 kV, 10 mA, 30 s)^47^. After 30 s, the excess of AuNP sample was removed with a filter paper, and the grid was air-dried at room temperature (RT) and then analyzed at TEM at 80 kV. AuNP size and analysis was performed using AnalySis 5.2 software (EMSIS, Muenster, Germany) and R free software, version 4.1.2 [R Core Team 2021]^48^.

#### Scanning electron microscopy (SEM) and energy dispersive X-ray spectroscopy (EDS)

Approximately 10 mm × 10 mm silicon wafer pieces were glow-discharge activated^47^. Immediately, 10 µL of AuNP dispersion was pipetted onto the activated wafer surface, and the wafers were dried at 56 °C for 4 h on a filter paper in a glass Petri dish. The wafers were then mounted on standard aluminum stubs with conductive silver paint (Leitsilber, Dr. Ropertz-GmbH, Munich, Germany) and baked overnight at 56 °C. Baked SEM stubs were coated with 10 nm of carbon in a high-resolution turbo-pumped Sputter Coater Q150T (Quorum Technologies Ltd, Ringmer, UK). SEM and energy-dispersive X-ray spectroscopy (EDS) were done in FEI Nova Nano SEM 450 scanning electron microscope (FEI, Brno, Czech Republic) equipped with an Ametek^®^ EDAX Octane plus SDD detector and TEAM EDS analysis systems (AMETEK B. V.; Tilburg, The Netherlands)^24^.

#### Size and zeta-potential analysis before conjugation with Nas

The average hydrodynamic size and zeta potential of AuNP were determined using Zetasizer Ultra (Malvern Panalytical, Malvern, UK). For size analysis, 50 µL of sample was added to an ultra-low volume ZEN2112 quartz cuvette and the signal was recorded in backscatter mode. For zeta potential analysis, 1 mL of the sample was added to a DTS1080 folded capillary zeta cell. The temperature was 25 °C and ddH_2_O was used as dispersant^49^.

#### Graphite furnace atomic absorption spectroscopy (GF-AAS)

The amount of AuNP before conjugation with NAs was determined by GF-AAS. 20 µL of sample was quantitatively transferred into a 50 mL polypropylene test tube containing freshly prepared 5 mL aqua regia (mixture of concentrated HNO_3_ and HCl in the ratio of 1:3 v/v) and allowed to react at room temperature for 6 h to dissolve the AuNP in the sample. The contents were then quantitatively transferred to a 100 mL volumetric flask and diluted to reach 20-fold diluted aqua regia and total Au content was determined^50^.

A set of matrix-matched (20-fold diluted aqua regia) calibration standards containing 0, 1, 3, 10, 30 and 100 ng mL^−1^ Au were prepared. GF-AAS (Perkin Elmer AAnalyst 800 with Zeeman background correction, USA), using the following experimental parameters: Au hollow cathode lamp operated at 242.8 nm, lamp current 10 mA, slit width 0.7 nm (L) A permanent modification of the graphite furnace was used^50^.

#### Conjugation of AuNP with NAs

Four different single- and double-stranded DNA and RNA molecules were used for the analysis. DNA samples were: (1) a ssDNA oligo 5′-CAA TAG TGA TGA CCT GGC CGT-3′ labelled at 5′ with Alexa Fluor 488 and (2) the sequence of dsDNA sense strand 5′-TGC ACC ACC AAC TGC TTA G-3′ duplexed with antisense strand 5′-CTAAGCAGTTGGTGGTGCA-3′. RNA samples were (1) ssRNA 5′ rUrCrA rCrArU rArGrG rArArU rGrArA rArArG rCrCrA rUrA-3′ labelled at 5′ with Alexa Fluor 488; this ssRNA has been designed specifically as inhibitory sequence of microRNA-135b (antimiR-135b)^20^ and (2) dsRNA consisting of sense strand 5′**-**rGrCrC rUrArU rUrUrC rUrGrC rCrArU rGrGrC rArArA AA-3′ duplexed with antisense strand: 5′**-**rUrUrU rGrCrC rArUrG rGrCrA rGrArA rArUrA rGrGrC TT-3′. To obtain the best conjugates, different concentrations of each NA were analyzed in diluted AuNP solution (with OD below 1) and after evaluation by electrophoresis, the best ones were used for in vitro experiment. Briefly, each NA (100 µmol) was dissolved in an amount of AuNP to obtain final concentrations of 2.5, 5, and 10 µmol and a final volume of 0.25 mL. The mixtures were incubated in a thermomixer with 0.5 mL thermoblock (Eppendorf, Hamburg, Germany) at 1600 rpm in a cold room at 4 °C overnight^36,37^. The next day, the mixtures were washed twice following the same protocol mentioned earlier. The pellet of each sample was dissolved in 50 µL RNase-free ddH_2_O and used for further analyses.

### Proof of AuNP conjugation with NAs

#### Visible spectrophotometry for AuNP-NAs

The changes in the maximum absorption peaks after conjugation of NA molecules to AuNP were analyzed using spectrophotometer. The changes in the peak area correspond to the binding of the AuNP to the NA molecules. The blank was ddH_2_O and all samples were diluted twice^38^.

#### Agarose electrophoresis

Agarose gel electrophoresis was used for three different concentrations of each AuNP-ssRNA, AuNP-dsRNA, AuNP-ssDNA, and AuNP-dsDNA conjugates. For this purpose, 2% w/v agarose (Sigma Aldrich, Prague, Czech Republic) containing GelRed™ nucleic acid stain (Biotium, California, USA), in Tris–acetate-EDTA buffer (TAE) was used and the gel ran under voltage of 110 V for 40 min. 5 µL of each sample was added to the wells after mixing with 1 µL of 6X MassRuler DNA loading dye (Thermo Fisher Scientific, Massachusetts, USA). Controls were MassRuler DNA ladder (Thermo Fisher Scientific, Prague, Czech Republic), AuNP, and four different free (non-conjugated) NAs: ssRNA, dsRNA, ssDNA, and dsDNA. When the NAs were conjugated to the AuNP, there was a migration delay in the conjugates in contrast to the control. The delayed migration of the NAs proves the successful conjugation^51,52^.

#### Fourier-transform infrared spectroscopy (FTIR)

AuNP as control and in conjugation with four different NAs (i.e. AuNP-ssRNA, AuNP-dsRNA, AuNP-ssDNA, and AuNP-dsDNA) were analyzed by FTIR. The spectra of AuNP and AuNP-NAs water dispersions were recorded using Vertex 70v FTIR spectrometer (Brucker Optics GmbH, Germany) in a BioATRII cell with ZnSe crystal in absorption mode. The spectra were recorded in the MIR range between 900 and 4000 cm^−1^ against ddH_2_O background at 25 ºC with a resolution of 4 cm^−1^, each spectrum representing the average of 128–300 scans. The different spectra obtained were compared with the FTIR spectrum of pure AuNP^38^.

#### Zetasizer analysis after conjugation with NAs

The zeta potential and size distribution of four different conjugates (i.e. AuNP-ssRNA, AuNP-dsRNA, AuNP-ssDNA, and AuNP-dsDNA) compared with AuNP as control were determined using the same technique as described previously^49^.

#### In vitro analysis

Prior to in vitro analysis, AuNP were first sterilized using the Tyndallization method and then used for the experiments. For this purpose, the washed AuNP were incubated in the steam of boiling water bath for 30 min and the microtube was placed at RT for 24 h. This procedure was repeated three times and the sterility of AuNP was checked by spreading a loop of AuNP on the surface of Sabouraud Dextrose Agar (SDA, Sigma Aldrich, Prague, Czech Republic) and Nutrient Agar (NA, Sigma Aldrich, Prague, Czech Republic) plates and incubated at 30 °C and 37 °C for 5 days and 24 h, respectively^53^.

#### MTT assay

Two different cell lines were used in this study: NIH/3T3 (ATCC CRL-1658) and 4T1 (ATCC CRL-2539). The first served as a control and was a normal fibroblast cell line from NIH-Swiss mice and the second was a breast cancer cell line from the BALB/c mouse strain. Dulbecco's Modified Eagle Medium (DMEM, Sigma Aldrich, Prague, Czech Republic) plus 10% fetal bovine serum (FBS, Gibco, Massachusetts, USA) and 44 µg/mL gentamicin (Sandoz, Novartis Company, Prague, Czech Republic) were used to culture NIH/3T3 cells. Roswell Park Memorial Institute Medium 1640 (RPMI-1640, Sigma Aldrich, Prague, Czech Republic) plus 10% FBS, 44 µg/mL gentamicin and 4.5 g/L glucose (Sigma Aldrich, Prague, Czech Republic) was used for culturing 4T1 cells. In order to have cells in exponential growth phase, cells were subcultured thrice a week. The MTT assay was performed to evaluate if AuNP trigger any cell toxicity in vitro. The two cell lines were analyzed separately. We used 96 flat-bottom tissue culture plates (JETBiofil, Guangzhou, China), added 2.5 × 10^5^ cells/well to each well in all rows of the plate and incubated overnight in a humid atmosphere, 5% CO_2_ incubator at 37 °C to achieve 80% confluency. The next day, cell medium was removed and all wells were replenished with 200 µL of fresh medium. We used 50 µL of the washed and sterilized AuNP at the highest concentration and its serial dilution using the ½-titration technique. We also considered a control sample without addition of AuNP. The samples were prepared and measured in independent triplicates. The plate was incubated overnight and the next day 20 µL of 5 mg/mL (3-4,5-dimethylthiozol-2-yl) 2,5-diphenyltetrazolium bromide (MTT, EMD Millipore, CA, USA) in phosphate buffered saline (pH 7.4) dye solution was added to all wells and incubated in a humid atmosphere, 5% CO_2_ incubator at 37 °C for 4 h. Then the mixture was removed and 200 µL of dimethyl sulfoxide (DMSO, Sigma Aldrich, Prague, Czech Republic) was added to the wells. After mixing, the absorbance of the wells was measured using a Tecan spectrophotometer (Thermo Fisher Scientific, Prague, Czech Republic) at a test wavelength of 570 nm and a reference wavelength of 630 nm. Finally, the percentage of half-maximal inhibitory concentration (IC_50_) of AuNP for each cell line was determined using the following formula^53^:

$$\begin{aligned} {\text{IC}}_{{{5}0}} & = {\text{OD of the maximal inhibition}} \hfill \\ & \quad - {5}0\% \, \left( {{\text{OD of the maximal inhibition}}\, - \,{\text{OD of the minimal inhibition}}} \right). \hfill \\ \end{aligned}$$

#### Confocal microscopy

For confocal microscopy, we used ssRNA and ssDNA samples that were labeled with Alexa Fluor 488 dye. Both NIH/3T3 and 4T1 cells in their exponential growth phase were incubated with the non-toxic (determined by the MTT assay) concentration of AuNP-Alexa-ssRNA and AuNP-Alexa-ssDNA conjugates. The penetration of the conjugates into particular cells was analyzed under a confocal microscope after 30 min and 24 h of incubation. For this analysis, cells were seeded on a 35-mm plate with a 20-mm glass bottom (Cellvis, Ontario, Canada) in concentration of 2.5 × 10^5^ cells/plate. Culture and incubation conditions were the same as for the MTT assay. After 80% confluence of cells was achieved, medium was replaced with 1500 µL of fresh medium/plate and 50 µL of particular conjugate. This conjugate concentration was determined from the results of the MTT assay. All plates were incubated in a humid atmosphere, 5% CO_2_ and 37 °C. Two plates were fixed after 30 min and other two after 24 h. After that cells were stained and examined under confocal microscope^20^.

For fixation, cells were washed twice with PBS and 500 µL of 4% paraformaldehyde was added to each plate. After 20 min, the fixation mixture was removed and the cells were washed twice with PBS. Cells were incubated with 0.1% Triton X-100 for 5 min and then washed twice. Phalloidin conjugate (CytoPainter F-actin Staining Kit, Abcam, Cambridge, UK) was used to stain the cytoskeleton, and 0.6 µL of the dye was added to 600 µL of PBS. After 30 min of incubation, the dye was discarded and the cells were washed twice with PBS. Hoechst33342 (1 µg/mL, Invitrogen, Massachusetts, USA) was used to stain the nuclei. Cells were incubated with 100 µL Hoechst 33342 (Invitrogen, Life Technologies, Prague, Czech Republic) at the final concentration of 1 µg/mL and immediately analyzed under Olympus FluoView FV1000 confocal microscope (objectives 20×/NA 0.75 and 40×/NA 0.95). Conjugates were detected due to Alexa Fluor 488 linked to the nucleic acid cargo. The excitation/emission parameters were as follows: Hoechst 405/461 nm; Alexa Fluor 488 473/520 nm; and Phalloidin 550/572 nm. Finally, the obtained results were analyzed using Olympus FluoView 2.0 software^20^.

### Quantitative real-time-polymerase chain reaction (qPCR) analysis

#### Samples preparation

To evaluate release of ssRNA cargo from AuNP we employed inhibitory ssRNA sequence (antimiR-135b)^20^ specifically targeting microRNA-135b (miR-135b) that is produced in high amount by cancer cells 4T1. Once the antimiR-135b is released from AuNP and is present in cell cytoplasm, it will form duplex with target microRNA-135b produced by cells and the duplex will initiate target RNA degradation. Thus, if the AuNP-mediated ssRNA transfection and its release is successful, the level of target microRNA-135b should be reduced. The cytoplasmic level of microRNA-135b was examined by qPCR. In parallel, control transfection samples with equal load of ssRNA (10 µmol) were prepared: AuNP-PEI-ssRNA and HP-ssRNA. For the preparation of AuNP-PEI-ssRNA, 200 µL AuNP were mixed with 200 µL of low weight PEI 800 (Sigma-Aldrich, Prague, Czech Republic; 0.9 mg/mL) and incubated in a thermomixer at 1600 rpm in a cold room at 4 °C overnight. The next day, the dispersion was centrifuged at 9000 rcf for 60 min and the pellet was dispersed in 200 µL RNase free ddH_2_O. Then, 20 µL of antimiR (100 µmol) was mixed with the AuNP-PEI and incubated overnight in a thermomixer at 4 °C. The dispersion was centrifuged and washed three times; the pellet was dispersed in 200 µL RNase-free ddH_2_O and 40 µL of the ssRNA-AuNP-PEI sample was added for every 1 mL of cell culture. To prepare HP-ssRNA, as a control without AuNP, X-tremeGENE HP DNA Transfection Reagent (Roche, Prague, Czech Republic) was used for transfection instead of AuNP. The manufacture’s protocol was followed: 20 µL of antimiR-135b (100 µmol) was mixed with 13.2 µL of HP and 186.8 µL RNase-free ddH_2_O, vortexed well, and incubated in room temperature for 30 min prior to cells transfection. 40 µL of the HP-ssRNA sample was added for every 1 mL of cell culture.

#### Cell culture and transfection

4T1 cells in their exponential growth phase were cultured in a 6-well plate. The medium and cell culture technique used were the same as for the MTT assay. The 50% confluent monolayer cells were washed with PBS and 1 mL of fresh working medium was added to each of the wells. 40 µL of each sample, AuNP-ssRNA, HP-ssRNA and AuNP-PEI-ssRNA, were added separately to each well. One well was left unchanged and served as control, and the plate was incubated overnight as for MTT and confocal microscopy assays.

#### microRNA isolation

After overnight incubation, cells were washed with PBS and microRNAs were isolated using a High Pure miRNA isolation Kit (Roche, Prague, Czech Republic) according to the manufacturer's instructions^20^. The yield of microRNA was determined using Invitrogen Qubit RNA high sensitivity (HS) Kit (Thermo Fisher Scientific, Prague, Czech Republic).

#### cDNA synthesis

For cDNA synthesis, the High Capacity cDNA Reverse Transcription Kit (Thermo Fisher Scientific, Prague, Czech Republic) was used. The starting concentration of microRNA was about 75 ng per reaction. Primers were specific reverse transcription primers for miR-135b (assay MI0000810) and miR-16 (assay MI0000070) (Thermo Fisher Scientific, Prague, Czech Republic). The procedure was performed according to the manufacturer's instructions.

#### qPCR

For qPCR, TaqMan Universal PCR Master Mix (No AmpErase) and miR-specific PCR primers (miR-135b assay MI0000810 and miR-16 assay MI0000070) were used and triplicates were performed for each sample in the iQ5 Real Time PCR Detection System (BioRad, Prague, Czech Republic). The results were analyzed using iQ5 Optical System Software 2.1 (BioRad, Prague, Czech Republic) and the expression of miR-135b was normalized to the expression of the internal control (miR-16)^20^.

### Statistical analyses

#### Biological assays

If not stated otherwise, we obtained data from three experiments, and presented them as averages and standard deviations. We used ANOVA/post-hoc Tukey test for group comparison (online tool available at http://astatsa.com/OneWay_Anova_with_TukeyHSD/). Values of p ≤ 0.05 (*) and p ≤ 0.01 (**) were considered as statistically significant.

#### TEM analyses

AuNP size and analysis was performed using AnalySis 5.2 software (EMSIS, Muenster, Germany) and R free software, version 4.1.2 [R Core Team 2021]^48^.

### Supplementary Information

Supplementary Information.

## Data Availability

The data supporting the results of the current study are available in the article file. All other data are available from the corresponding author upon request.

## References

[1] Pourali P (2020). Histopathological study of the maternal exposure to the biologically produced silver nanoparticles on different organs of the offspring. Naunyn Schmiedebergs Arch. Pharmacol..

[2] Hainfeld JF (2014). Gold nanoparticle hyperthermia reduces radiotherapy dose. Nanomed. Nanotechnol. Biol. Med..

[3] Arvizo R, Bhattacharya R, Mukherjee P (2010). Gold nanoparticles: Opportunities and challenges in nanomedicine. Expert Opin. Drug Deliv..

[4] Nel A, Xia T, Mädler L, Li N (2006). Toxic potential of materials at the nanolevel. Science.

[5] Pourali P (2017). Biosynthesis of gold nanoparticles by two bacterial and fungal strains, *Bacillus cereus* and *Fusarium oxysporum*, and assessment and comparison of their nanotoxicity in vitro by direct and indirect assays. Electron. J. Biotechnol..

[6] Graczyk A, Pawlowska R, Jedrzejczyk D, Chworos A (2020). Gold nanoparticles in conjunction with nucleic acids as a modern molecular system for cellular delivery. Molecules.

[7] McIntosh CM (2001). Inhibition of DNA transcription using cationic mixed monolayer protected gold clusters. J. Am. Chem. Soc..

[8] Han G, Martin CT, Rotello VM (2006). Stability of gold nanoparticle-bound DNA toward biological, physical, and chemical agents. Chem. Biol. Drug Des..

[9] Bonoiu AC (2009). Nanotechnology approach for drug addiction therapy: Gene silencing using delivery of gold nanorod-siRNA nanoplex in dopaminergic neurons. Proc. Natl. Acad. Sci..

[10] Aali E, Shokuhi Rad A, Esfahanian M (2020). Computational investigation of the strategy of DNA/RNA stabilization through the study of the conjugation of an oligonucleotide with silver and gold nanoparticles. Appl. Organomet. Chem..

[11] Huang C-C, Huang Y-F, Cao Z, Tan W, Chang H-T (2005). Aptamer-modified gold nanoparticles for colorimetric determination of platelet-derived growth factors and their receptors. Anal. Chem..

[12] Cho H (2008). Aptamer-based SERRS sensor for thrombin detection. Nano Lett..

[13] Medley CD (2008). Gold nanoparticle-based colorimetric assay for the direct detection of cancerous cells. Anal. Chem..

[14] Mastroianni AJ, Claridge SA, Alivisatos AP (2009). Pyramidal and chiral groupings of gold nanocrystals assembled using DNA scaffolds. J. Am. Chem. Soc..

[15] Lee J-S (2009). Gold, poly(β-amino ester) nanoparticles for small interfering RNA delivery. Nano Lett..

[16] Mendes R, Fernandes AR, Baptista PV (2017). Gold nanoparticle approach to the selective delivery of gene silencing in cancer—the case for combined delivery?. Genes.

[17] Sandhu KK, McIntosh CM, Simard JM, Smith SW, Rotello VM (2002). Gold nanoparticle-mediated transfection of mammalian cells. Bioconjug. Chem..

[18] Thomas M, Klibanov AM (2003). Conjugation to gold nanoparticles enhances polyethylenimine's transfer of plasmid DNA into mammalian cells. Proc. Natl. Acad. Sci..

[19] Javed R (2020). Role of capping agents in the application of nanoparticles in biomedicine and environmental remediation: Recent trends and future prospects. J. Nanobiotechnol..

[20] Křivohlavá R, Neuhӧferová E, Jakobsen KQ, Benson V (2019). Knockdown of microRNA-135b in mammary carcinoma by targeted nanodiamonds: Potentials and pitfalls of in vivo applications. Nanomaterials.

[21] Borchard G (2001). Chitosans for gene delivery. Adv. Drug Deliv. Rev..

[22] Martin ME, Rice KG (2007). Peptide-guided gene delivery. AAPS J..

[23] Maguire CM, Rösslein M, Wick P, Prina-Mello A (2018). Characterisation of particles in solution—A perspective on light scattering and comparative technologies. Sci. Technol. Adv. Mater..

[24] Pourali P (2021). Response of biological gold nanoparticles to different pH values: Is it possible to prepare both negatively and positively charged nanoparticles?. Appl. Sci..

[25] Narayanan KB, Sakthivel N (2011). Facile green synthesis of gold nanostructures by NADPH-dependent enzyme from the extract of Sclerotium rolfsii. Colloids Surf. A.

[26] Narayanan KB, Sakthivel N (2010). Biological synthesis of metal nanoparticles by microbes. Adv. Coll. Interface. Sci..

[27] McVey C, Huang F, Elliott C, Cao C (2017). Endonuclease controlled aggregation of gold nanoparticles for the ultrasensitive detection of pathogenic bacterial DNA. Biosens. Bioelectron..

[28] Shawky SM, Bald D, Azzazy HM (2010). Direct detection of unamplified hepatitis C virus RNA using unmodified gold nanoparticles. Clin. Biochem..

[29] Li H, Rothberg LJ (2004). Label-free colorimetric detection of specific sequences in genomic DNA amplified by the polymerase chain reaction. J. Am. Chem. Soc..

[30] Li H, Rothberg L (2004). Colorimetric detection of DNA sequences based on electrostatic interactions with unmodified gold nanoparticles. Proc. Natl. Acad. Sci..

[31] Giljohann DA, Seferos DS, Prigodich AE, Patel PC, Mirkin CA (2009). Gene regulation with polyvalent siRNA−nanoparticle conjugates. J. Am. Chem. Soc..

[32] Lytton-Jean AK, Langer R, Anderson DG (2011). Five years of siRNA delivery: Spotlight on gold nanoparticles. Small.

[33] Oishi M, Nakaogami J, Ishii T, Nagasaki Y (2006). Smart PEGylated gold nanoparticles for the cytoplasmic delivery of siRNA to induce enhanced gene silencing. Chem. Lett..

[34] Ghosh PS (2007). Nanoparticles featuring amino acid-functionalized side chains as DNA receptors. Chem. Biol. Drug Des..

[35] Pourali P, Neuhöferová E, Dzmitruk V, Benson V (2022). Investigation of protein corona formed around biologically produced gold nanoparticles. Materials.

[36] Pourali P, Yahyaei B, Afsharnezhad S (2018). Bio-synthesis of gold nanoparticles by *Fusarium oxysporum* and assessment of their conjugation possibility with two types of β-lactam antibiotics without any additional linkers. Microbiology.

[37] Naimi-Shamel N, Pourali P, Dolatabadi S (2019). Green synthesis of gold nanoparticles using *Fusarium oxysporum* and antibacterial activity of its tetracycline conjugant. Journal de mycologie medicale.

[38] Yahyaei B, Pourali P (2019). One step conjugation of some chemotherapeutic drugs to the biologically produced gold nanoparticles and assessment of their anticancer effects. Sci. Rep..

[39] Pourali P (2023). Fate of the capping agent of biologically produced gold nanoparticles and adsorption of enzymes onto their surface. Sci. Rep..

[40] Yahyaei B, Nouri M, Bakherad S, Hassani M, Pourali P (2019). Effects of biologically produced gold nanoparticles: Toxicity assessment in different rat organs after intraperitoneal injection. AMB Express.

[41] Fratoddi I, Venditti I, Cametti C, Russo MV (2015). How toxic are gold nanoparticles? The state-of-the-art. Nano Res..

[42] Ding Y (2014). Gold nanoparticles for nucleic acid delivery. Mol. Ther..

[43] Krokan, H. E., Kavli, B., Slupphaug, G. & Drabløs, F. In *Genomic Uracil: Evolution, Biology, Immunology and Disease* 15–45 (World Scientific, 2018).

[44] Walter W, Stein U (2000). Viral vectors for gene transfer a review of their use in the treatment of human disease. Drugs.

[45] Kay MA, Glorioso JC, Naldini L (2001). Viral vectors for gene therapy: The art of turning infectious agents into vehicles of therapeutics. Nat. Med..

[46] Takahashi Y, Nishikawa M, Takakura Y (2009). Nonviral vector-mediated RNA interference: Its gene silencing characteristics and important factors to achieve RNAi-based gene therapy. Adv. Drug Deliv. Rev..

[47] Benada O, Pokorný V (1990). Modification of the Polaron sputter-coater unit for glow-discharge activation of carbon support films. J. Electron Microsc. Tech..

[48] Team, R. C. R: A language and environment for statistical computing. (2021).

[49] Park S (2019). Reversibly pH-responsive gold nanoparticles and their applications for photothermal cancer therapy. Sci. Rep..

[50] Welz B, Sperling M (2008). Atomic Absorption Spectrometry.

[51] Wang H-Q, Deng Z-X (2015). Gel electrophoresis as a nanoseparation tool serving DNA nanotechnology. Chin. Chem. Lett..

[52] Ackerson CJ, Sykes MT, Kornberg RD (2005). Defined DNA/nanoparticle conjugates. Proc. Natl. Acad. Sci..

[53] Pourali P, Yahyaei B (2016). Biological production of silver nanoparticles by soil isolated bacteria and preliminary study of their cytotoxicity and cutaneous wound healing efficiency in rat. J. Trace Elem. Med Biol..

