# Supplementary material for: Conjugation of microbial-derived gold nanoparticles to different types of nucleic acids: evaluation of transfection efficiency
Source: Sci Rep. 2023 Sep 6;13:14669. doi: 10.1038/s41598-023-41567-7 (PMC10482973; doi:10.1038/s41598-023-41567-7)
Supplement: Supplementary file 1 — Supplementary Information. [file 41598_2023_41567_MOESM1_ESM.docx]

Supplementary Table 1. The visible absorbance values of AuNPs in contrast to AuNPs-ssRNA and AuNPs-dsRNA.

| **Wavelength (nm)** | **OD for AuNPs** | **OD for AuNPs-ssRNA** | **OD for AuNPs-dsRNA** |
| --- | --- | --- | --- |
| 525 | 0.568 | 0.761 | 0.978 |
| 526 | 0.569 | 0.766 | 0.985 |
| 527 | 0.57 | 0.77 | 0.992 |
| 528 | 0.572 | 0.773 | 0.997 |
| 529 | 0.574 | 0.773 | 0.999 |
| 530 | 0.575 | 0.771 | 0.995 |
| 531 | 0.575 | 0.771 | 0.994 |
| 532 | 0.576 | 0.773 | 0.994 |
| 533 | 0.577 | 0.774 | 0.995 |
| 534 | 0.576 | 0.773 | 0.998 |
| 535 | 0.575 | 0.774 | 0.999 |
| 536 | 0.572 | 0.774 | 0.995 |
| 537 | 0.57 | 0.772 | 0.988 |
| 538 | 0.569 | 0.77 | 0.986 |
| 539 | 0.57 | 0.77 | 0.984 |
| 540 | 0.567 | 0.77 | 0.985 |
| 541 | 0.564 | 0.77 | 0.979 |
| 542 | 0.56 | 0.769 | 0.971 |
| 543 | 0.558 | 0.766 | 0.964 |
| 544 | 0.554 | 0.763 | 0.957 |
| 545 | 0.549 | 0.761 | 0.951 |
| 546 | 0.546 | 0.757 | 0.941 |
| 547 | 0.543 | 0.752 | 0.935 |
| 548 | 0.54 | 0.748 | 0.926 |
| 549 | 0.535 | 0.745 | 0.92 |
| 550 | 0.53 | 0.74 | 0.908 |
| 551 | 0.524 | 0.734 | 0.898 |
| 552 | 0.519 | 0.728 | 0.889 |
| 553 | 0.51 | 0.723 | 0.882 |
| 554 | 0.504 | 0.72 | 0.874 |
| 555 | 0.498 | 0.713 | 0.857 |
| 556 | 0.494 | 0.71 | 0.844 |
| 557 | 0.488 | 0.706 | 0.832 |
| 558 | 0.482 | 0.702 | 0.822 |
| 559 | 0.476 | 0.696 | 0.814 |
| 560 | 0.469 | 0.684 | 0.799 |

Supplementary Table 2. The visible absorbance values of AuNPs in contrast to AuNPs-ssDNA and AuNPs-dsDNA.

| \| **Wavelength (nm)** \| **OD for AuNPs** \| **OD for AuNPs-ssDNA** \| **OD for AuNPs-dsDNA** \| \| --- \| --- \| --- \| --- \| \| 525 \| 0.568 \| 0.481 \| 0.433 \| \| 526 \| 0.569 \| 0.484 \| 0.436 \| \| 527 \| 0.57 \| 0.487 \| 0.439 \| \| 528 \| 0.572 \| 0.489 \| 0.442 \| \| 529 \| 0.574 \| 0.49 \| 0.443 \| \| 530 \| 0.575 \| 0.489 \| 0.443 \| \| 531 \| 0.575 \| 0.487 \| 0.443 \| \| 532 \| 0.576 \| 0.487 \| 0.447 \| \| 533 \| 0.577 \| 0.488 \| 0.45 \| \| 534 \| 0.576 \| 0.49 \| 0.451 \| \| 535 \| 0.575 \| 0.488 \| 0.45 \| \| 536 \| 0.572 \| 0.488 \| 0.452 \| \| 537 \| 0.57 \| 0.488 \| 0.455 \| \| 538 \| 0.569 \| 0.488 \| 0.456 \| \| 539 \| 0.57 \| 0.487 \| 0.459 \| \| 540 \| 0.567 \| 0.486 \| 0.462 \| \| 541 \| 0.564 \| 0.484 \| 0.463 \| \| 542 \| 0.56 \| 0.48 \| 0.46 \| \| 543 \| 0.558 \| 0.476 \| 0.458 \| \| 544 \| 0.554 \| 0.475 \| 0.458 \| \| 545 \| 0.549 \| 0.474 \| 0.461 \| \| 546 \| 0.546 \| 0.472 \| 0.463 \| \| 547 \| 0.543 \| 0.469 \| 0.463 \| \| 548 \| 0.54 \| 0.466 \| 0.461 \| \| 549 \| 0.535 \| 0.461 \| 0.459 \| \| 550 \| 0.53 \| 0.457 \| 0.461 \| \| 551 \| 0.524 \| 0.454 \| 0.461 \| \| 552 \| 0.519 \| 0.453 \| 0.462 \| \| 553 \| 0.51 \| 0.45 \| 0.462 \| \| 554 \| 0.504 \| 0.448 \| 0.463 \| \| 555 \| 0.498 \| 0.444 \| 0.464 \| \| 556 \| 0.494 \| 0.441 \| 0.463 \| \| 557 \| 0.488 \| 0.435 \| 0.461 \| \| 558 \| 0.482 \| 0.432 \| 0.458 \| \| 559 \| 0.476 \| 0.427 \| 0.458 \| \| 560 \| 0.469 \| 0.42 \| 0.457 \| |  |  |  |
| --- | --- | --- | --- | --- | --- | --- | --- | --- | --- | --- | --- | --- | --- | --- | --- | --- | --- | --- | --- | --- | --- | --- | --- | --- | --- | --- | --- | --- | --- | --- | --- | --- | --- | --- | --- | --- | --- | --- | --- | --- | --- | --- | --- | --- | --- | --- | --- | --- | --- | --- | --- | --- | --- | --- | --- | --- | --- | --- | --- | --- | --- | --- | --- | --- | --- | --- | --- | --- | --- | --- | --- | --- | --- | --- | --- | --- | --- | --- | --- | --- | --- | --- | --- | --- | --- | --- | --- | --- | --- | --- | --- | --- | --- | --- | --- | --- | --- | --- | --- | --- | --- | --- | --- | --- | --- | --- | --- | --- | --- | --- | --- | --- | --- | --- | --- | --- | --- | --- | --- | --- | --- | --- | --- | --- | --- | --- | --- | --- | --- | --- | --- | --- | --- | --- | --- | --- | --- | --- | --- | --- | --- | --- | --- | --- | --- | --- | --- | --- | --- | --- | --- |

Supplementary Table 3. The absorbance values measured with MTT assay in NIH/3T3 and 4T1 cell lines. A test wavelength was 570 nm and a reference wavelength was 630 nm. Rows A-D contained NIH/3T3 and rows E-H contained 4T1 cell lines, respectively. Each line contained sterilized AuNPs at the highest concentration and its serial dilution using the ½-titration technique. 12^th^ well from each row is control without addition of AuNPs.

| <> | 1 | 2 | 3 | 4 | 5 | 6 | 7 | 8 | 9 | 10 | 11 | 12 |
| --- | --- | --- | --- | --- | --- | --- | --- | --- | --- | --- | --- | --- |
| A | 2.0476 | 2.8376 | 2.7669 | 2.3172 | 2.577 | 2.1232 | 2.7763 | 2.0889 | 2.243 | 2.1181 | 2.3386 | 1.885 |
| B | 2.6971 | 2.7352 | 2.0637 | 2.3783 | 2.2355 | 2.5127 | 2.1408 | 2.6414 | 2.3761 | 2.8223 | 2.6933 | 2.8457 |
| C | 2.9411 | 2.8192 | 2.4842 | 2.2878 | 2.6735 | 2.7919 | 2.5205 | 2.4728 | 2.0111 | 3.0879 | 2.766 | 2.5085 |
| D | 2.6737 | 2.6721 | 2.7939 | 2.7681 | 2.4287 | 2.7888 | 2.7986 | 2.6957 | 2.1348 | 2.1696 | 2.986 | 2.608 |
| E | 2.3283 | 2.5993 | 1.991 | 2.8845 | 2.3701 | 2.002 | 2.0791 | 2.0748 | 2.4939 | 1.9125 | 2.0717 | 2.6418 |
| F | 2.9321 | 2.1294 | 2.7009 | 2.4034 | 1.9909 | 2.1924 | 2.3003 | 2.2794 | 2.5778 | 2.3195 | 2.8416 | 2.8959 |
| G | 2.0131 | 2.4066 | 2.3962 | 1.9103 | 2.1428 | 2.1384 | 2.3481 | 1.9589 | 2.1822 | 1.8348 | 2.7786 | 2.9437 |
| H | 2.5932 | 2.1664 | 2.4434 | 2.6994 | 2.4478 | 2.4294 | 1.9576 | 1.9983 | 2.0456 | 1.9002 | 2.05 | 2.5744 |

Supplementary Fig 1. The obtained MTT assay plate for NIH/3T3 and 4T1 cell lines that corresponds to the data in Supplementary Table 3. Rows A-D contained NIH/3T3 and rows E-H contained 4T1 cell lines, respectively.


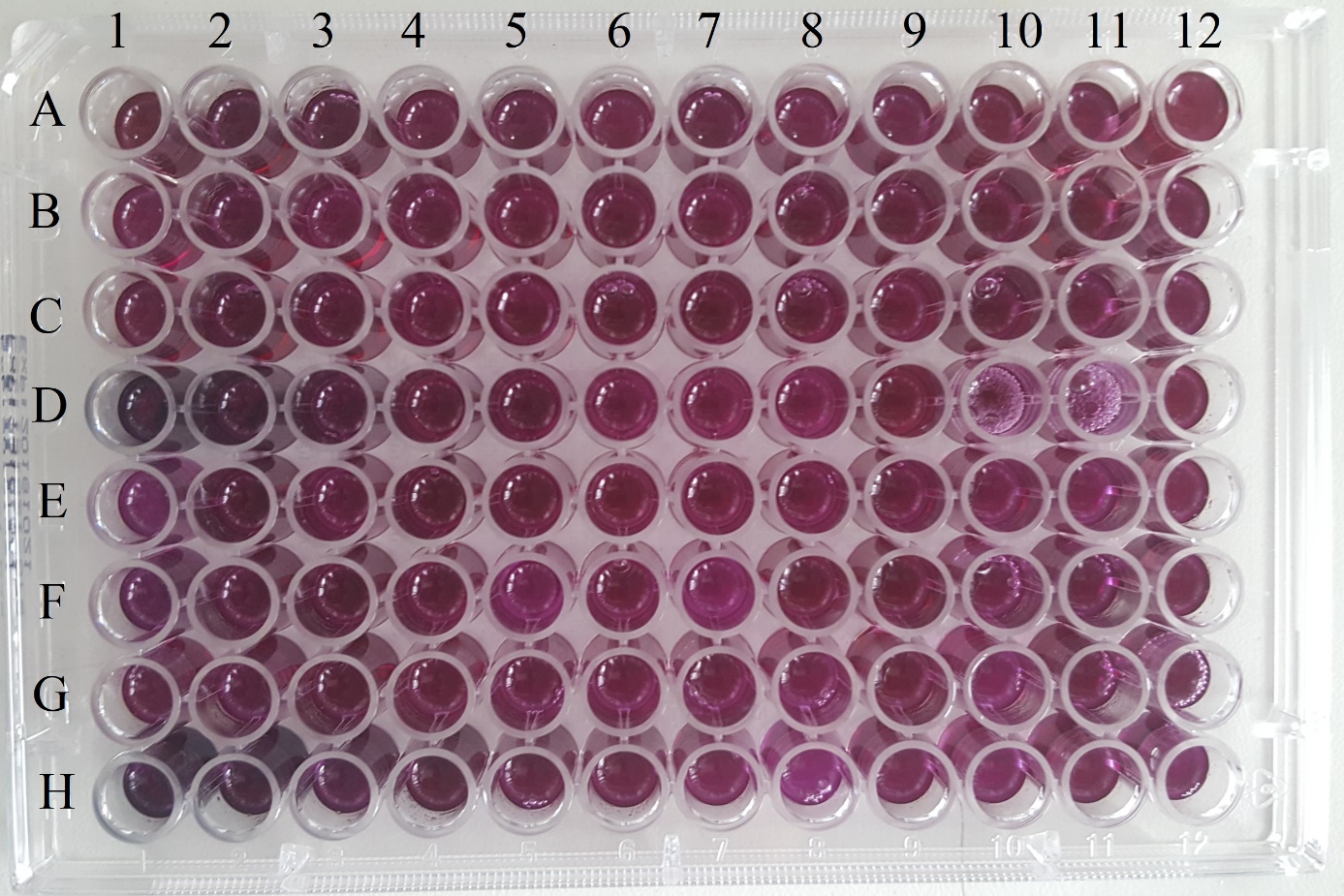


Supplementary Fig. 2 Relative quantities of miR-16 detected in different samples with a real-time PCR. The miR-16 quantities (arbitrary units) were counted with an iQ5 Optical System Software 2.1 from three different measurements and used for subsequent normalization of the target miR-135b levels.
